# Change in sedative burden after dementia onset using difference-in-difference estimations

**DOI:** 10.1371/journal.pone.0220582

**Published:** 2019-08-02

**Authors:** Young-Mi Ah, Euna Han, Kwanghee Jun, Yun Mi Yu, Ju-Yeun Lee

**Affiliations:** 1 College of Pharmacy, Yeungnam University, Gyeongsangbuk-do, Republic of Korea; 2 College of Pharmacy, Yonsei Institute for Pharmaceutical Research, Yonsei University, Incheon, Republic of Korea; 3 College of Pharmacy and Research Institute of Pharmaceutical Sciences, Seoul National University, Seoul, Republic of Korea; University of Malaya, MALAYSIA

## Abstract

**Background:**

Sedative agents are avoided in older adults because of potential risks including cognitive impairment, fall, frailty, and mortality. However, no studies addressing both prediagnostic and postdiagnostic period of dementia have evaluated sedative agent usage over an extended period.

**Objectives:**

To describe a longitudinal change in sedative medication use before and after the diagnosis with dementia over 10 years compared to patients without dementia.

**Methods:**

We conducted a retrospective cohort study using longitudinal claims data for senior national health insurance beneficiaries. After 1:4 propensity score matching, 54,165 older patients (≥60 years) were included. Difference-in-difference (DID) of sedative burden and use of sedative agents pre- and post-dementia diagnosis were estimated, and compared to those of patients without dementia. The yearly average daily sedative load (adSL) for each individual was calculated after applying duration, dose, and sedative score of medications from the sedative load model. The medication use for each sedative category was calculated using the defined daily dose (DDD) per 1000 patient-days.

**Results:**

The adSL in patients with dementia was consistently high before and after diagnosis and significantly increased after diagnosis, compared to those of patients without dementia (DID 0.123 unit/day, 95% confidence interval 0.117–0.129). DID of medication use was the highest for antidepressants (64.764 DDD/1000 patient-days) followed by Z-drugs and antipsychotics. Atypical antipsychotic and antidepressant usage steeply increased after dementia diagnosis.

**Conclusion:**

Sedative burden in patients with dementia before and after dementia diagnosis was higher than that in patients without dementia, and was further increased after dementia diagnosis.

## Introduction

Sedative effects are found in various medications including central nervous system (CNS) medications (hypnotics, sedatives, antipsychotics, antidepressants, and opioid analgesics), histamine H1 receptor antagonist, centrally acting muscle relaxants and some gastrointestinal medications [1]. The use of these medications in older adults is a well-known risk factor for adverse clinical outcomes such as impaired functional status, cognitive impairment, fall, frailty, and mortality [2–7]. Given such a high risk, the current clinical guidelines for medication use in older adults such as the Beers criteria and STOPP/START criteria recommend that the use of sedative agents such as long acting benzodiazepines and psychotropic medications should be avoided in older adults [8, 9]. However, these medications were being frequently used in older adults; 25–40% of home-dwelling older adults and 42–85% of institutionalized older adults were reported to use those sedative agents [10–13]. Elderly patients were also reported to have 6–12% of hospital admissions associated with adverse drug events, which could result from potentially inappropriate medications [14, 15]. Especially in patients with dementia, the use of antipsychotics is potentially inappropriate in older adults due to the elevated risk of stroke and mortality [8, 9]. Nevertheless, antipsychotics and hypnotics are frequently used in patients with dementia because of the clinical manifestations of dementia including sleep disturbance and behavioral and psychological symptoms. In a study conducted in the Europe, antipsychotics use in patients with dementia in long-term institutional care ranged from 12% in Sweden to 54% in Spain [16].

Prevalence and the economic burden of dementia is a global problem as the cost of dementia was estimated at 1.09% of the global GDP in 2015 [17]. The societal burden of disease regarding dementia is particularly one of major public health concerns in South Korea where the proportion of elderly patients (age≥65 years) has increased from 7.2% in 2000 to 14.2% in 2017 [18]. Therefore, an evaluation of the appropriateness of medication use in patients with dementia could be important to prevent adverse clinical outcomes and reduce socioeconomic burdens with regards to potentially inappropriate medications.

The use of sedative agents has been actively explored in the previous literature. However, most of the previous studies have been conducted in western countries and for selected patient groups according to residence type [19–22]. Furthermore, no studies have evaluated the use of sedative agents over a long period of time with addressed both the period before and after the diagnosis of dementia. This study builds on the previous literature and improves upon it by investigating a longitudinal change in sedative medication use over 10 years encompassing both the period before and after dementia diagnosis. We also established a pseudo-experimental control group without dementia using propensity matching to assess the incremental sedative use among patients with dementia. The current study extracted sedative agent use based on nationally representative real-world data.

## Methods

### Study design and data sources

This study was a retrospective cohort study using the Korea National Health Insurance Service Senior Cohort (KNHIS-SC) database (DB) which was based on insurance claims for older adults provided by National Health Insurance Service. National Health Insurance Service is the sole public health insurer in South Korea, with all legal residents in Korea as compulsory beneficiaries and all healthcare institutions including pharmacies as compulsory providers. KNHIS-SC DB is a longitudinal database for unidentified 558,147 seniors aged ≥ 60 years as of 2002, representing 10% of all Korean population of the same ages, who were followed-up until 2013. The dataset contains information on health care utilization, including screening services, mortality, and sociodemographic variables. This study was approved by the Yeungnam university institutional review board (YU 2019-01-001).

### Patient selection

We selected patients who were newly diagnosed with dementia (ICD-10 F00, F01, F02 and F03) and then initiated anti-dementia medications from 2003 to 2008. Patients who were never prescribed anti-dementia medications and were not diagnosed with dementia during 2003 and 2008 were selected as controls. The first year of dementia diagnosis was defined as index year. Propensity score matching method was applied to establish pseudo-experimental control group in order to reduce the effect of confounding factors. Age, gender, and co-morbid diseases (hypertension, diabetes mellitus, and dyslipidemia) were used in matching. Greedy matching was used, and controls were chosen to make sure the ratio of the case and control cohorts was 1:4 [23].

Exclusion criteria were as follows; 1) patients who died before 2010, whose minimum follow-up period was <2 years after the index year, 2) patients who had dementia diagnosis but were not prescribed anti-dementia medications, 3) patients who initiated anti-dementia medication before 2003 and after 2008.

### Variables

In order to evaluate the trend of sedative burden and sedative agent use before and after dementia diagnosis, we used two variables as dependent variables. First, we calculated each individual’s average daily sedative load (adSL) yearly for patients with prescription of systemic action agents during the study period using the below equation. To calculate sedative burden, we excluded patients who were prescribed only topical agents such as inhalers and eye drops because the systemic effect of those agents is estimated to be minimal. By using the following Eq (1), we could calculate the cumulative sedative burden that reflects the duration and dose of sedative agents. We then estimated the trends of adSL before and after diagnosis in patients with dementia compared to those without dementia.

$$Average\;Daily\;Sedative\;Load=\frac{\sum_{k=1}^{n}\frac{Total\;prescribed\;dose\;of\;Ak\;X\;SLMk}{WHO-DDD\;of\;Ak}}{365\;days}$$ (1)

Where A indicates the *k*th sedative agents prescribed to a patient (*k* = 1 to n); SLM, Sedative Load Model; WHO-DDD, defined daily dose by WHO. In the adSL equation, we used Sedative Load Model (SLM) to rank the degree of sedation. In this model, medications were categorized into four groups: group 1 (primary sedatives), group 2 (drugs with sedation as a prominent side effect), group 3 (drugs with sedation as a potential adverse side effect) and group 4 (drug with no known sedation). Each group was assigned sedative scores as follows; 2 for group 1, 1 for group 2, and 0 for group 3 and 4 [1, 20].

Second, we calculated DDD per 1000 patient-days for each sedative category before and after dementia diagnosis to identify the change in the medication use per each category of the sedative agents. Sedative category was classified on the basis of the original classification of a SLM. Subsequently, considering the pharmacological properties, the category of “Anxiolytics” and “Hypnotics and Sedatives” were modified for investigating the trends of medication use in detail. Modified medication categories were as follows; Benzodiazepines, z-drugs, other hypnotics and sedatives, and other anxiolytics (S1 Table).

The key independent variable was whether a patient was newly diagnosed with dementia in accordance with criteria for patient selection. We also identified the following baseline characteristics of each patient as covariates in order to control those variables in all estimations: age in years, sex [24], calendar year of medication prescription, and comorbidities including hypertension, diabetes mellitus, dyslipidemia, stroke, Parkinson’s disease, schizophrenia, and depression.

### Analysis

Multivariate log-normal mixed-effects regression was performed. Difference-in-difference in sedative burden between patients with dementia and patients without dementia adjusting the difference before the onset of dementia was estimated as in Eq (2). The group without dementia provided an estimate of the expected baseline change in the anti-sedative burden which was then subtracted from the change observed in the dementia group to estimate a net causal effect.

$$Y_{it}=\beta_{0}+\beta_{1}{Post}_{it}+\beta_{2}{Dementia}_{it}+\delta ({Dementia}_{it}\times {Post}_{it})+\beta_{3}X_{it}+\mu_{i}+\varepsilon_{it}$$ (2)

where the subscripts *i* and *t* denote individual and year, respectively. Y denotes drug exposure score. Post and Dementia are dummy indicators representing time after dementia onset (versus pre-diagnosis) and people with dementia (versus those without dementia), respectively. X is a vector of individual demographic and socioeconomic characteristics as covariates. Individual-level random effects were represented by *μ*. *ε* is a stochastic error term which was assumed for the data to be independently and identically distributed; *β* indicates parameters to be estimated. *β*_2_ is the incremental difference in drug exposure between patients with dementia and the controls before the diagnosis, and *β*_2_+δ is the corresponding difference after diagnosis. Therefore, δ is the parameter estimating difference-in-difference in the extent of the drug exposure between dementia and the control groups, which controls for pre-diagnosis differences between the two groups. The unit of analysis was individual-year. Standard errors were bootstrapped for robustness for all estimations.

Descriptive statistics such as mean, standard deviation (SD), and percentage were calculated. The chi-squared test was used to compare categorical variables, and the Student’s t-test was used to compare continuous variables between the two groups. We used SAS 9.4 (SAS Institute, Inc., Cary, NC, USA) and STATA (version 14; StataCorp LP, College Station, TX) for data management and statistical analysis.

## Results

**Fig 1:**
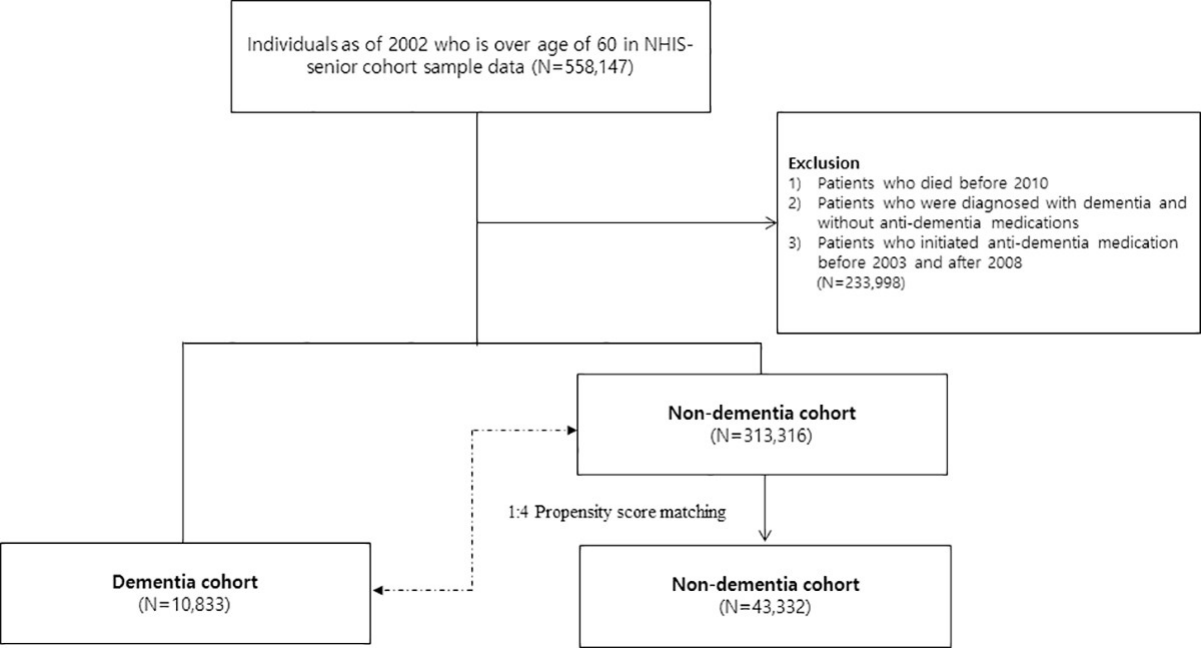
Flow of patient selection. NHIS; National Health Insurance Service.

Among the 558,147 seniors aged ≥ 60 years, patients with dementia were 10,833 and control patients without dementia were 313,316. After propensity score matching of 1:4 ratio, a final cohort of 54,165 patients was constructed (Fig 1).

The mean age was 71 years and approximately three-fourths of the cohorts were female. The prevalence of stroke (10.40% vs. 4.72%, p<0.001), Parkinson’s disease (1.44% vs. 0.21%, p<0.001), schizophrenia (0.57% vs. 0.06%, p <0.001), and depression (8.82% vs. 3.76%, p<0.001) were significantly higher in the dementia cohort than in the non-dementia cohort. Approximately one-third patients with dementia were first diagnosed in 2008. Medication use of both cohorts showed a similar tendency; a rapid decrease after reaching their peak in 2011. However, medication use in patients with dementia was higher than in patients without dementia during the overall study period (Table 1).

**Table 1:** Baseline characteristics of dementia and non-dementia cohort.

	Dementia (N = 10,833)	Non-Dementia (N = 43,332)	P-value
**Age, mean (SD)**	71.10 (6.42)	71.10 (6.44)	0.896
<75, N (%)	7,583 (70.00%)	30,328 (69.99%)	0.927
75~<85, N (%)	3,002 (27.71%)	11,985 (27.66%)	
85~, N (%)	248 (2.29%)	1,019 (2.35%)	
**Sex, Male, N (%)**	2,778 (25.64%)	11,115 (25.65%)	0.988
**Comorbidities, N(%)**			
Hypertension	4,070 (37.57%)	16,244 (37.49%)	0.873
Diabetes mellitus	1,122 (10.36%)	4,453 (10.28%)	0.805
Dyslipidemia	1,280 (11.82%)	5,011 (11.56%)	0.465
Stroke	1,127 (10.40%)	2,044 (4.72%)	<0.001
Parkinson	156 (1.44%)	90 (0.21%)	<0.001
Schizophrenia	62 (0.57%)	28 (0.06%)	<0.001
Depression	956 (8.82%)	1,629 (3.76%)	<0.001
**Index year, N(%)**			
2003	524 (4.8%)	2096 (4.8%)	-
2004	622 (5.7%)	2488 (5.7%)	
2005	1113 (10.3%)	4452 (10.3%)	
2006	1936 (17.9%)	7744 (17.9%)	
2007	2706 (25.0%)	10824 (25.0%)	
2008	3932 (36.3%)	15728 (36.3%)	
**Total patient-days of prescriptions per patient each year**			
2002	571	470	
2003	866	652	
2004	1071	782	
2005	1183	862	
2006	1624	1069	
2007	1901	1202	
2008	1906	1325	
2009	1959	1423	
2010	1904	1465	
2011	2013	1600	
2012	791	614	
2013	130	111	

The adjusted adSL of patients with dementia and patients without dementia after the index year were significantly increased than before index year, 0.135 and 0.012 respectively. After adjusting difference of patients without dementia, the adjusted adSL of dementia patients after diagnosis was still higher than before index year (0.123 unit/day, 95% confidence interval 0.117–0.129) (Table 2). After the index year, the proportion of patients that was prescribed sedative agents at least once in patients with and without dementia was 96.1% and 96.2%, respectively (S2 Table).

**Table 2:** The change in sedative agent use by medication group before and after diagnosis.

	Dementia		Non-dementia		Difference-in-difference			
	Δ estimate of difference of pre/post (SE)				Estimate (SE)		95% confidence interval	
**Adjusted average daily sedative load**								
	0.135	(0.004)*	0.012	(0.003)*	0.123	(0.003)*	0.117	0.129
**Adjusted average DDD / 1000 patient-days**											
** Antidepressants**								
SSRI etc.	64.828	(0.934)*	2.361	(0.742)*	62.467	(0.736)*	61.024	63.910
Tricyclic agents etc.	2.733	(0.343)*	0.440	(0.275)*	2.293	(0.268)*	1.767	2.818
** Z-drugs**	17.293	(0.656)*	-0.262	(0.521)	17.555	(0.518)*	16.540	18.570
** Antipsychotics**								
Atypical antipsychotics	14.710	(0.226)*	-0.652	(0.180)*	15.362	(0.178)*	15.013	15.712
Traditional antipsychotics	0.942	(0.154)*	0.120	(0.123)	0.822	(0.120)*	0.587	1.057
** Antiepileptics**	10.888	(0.462)*	0.344	(0.369)	10.544	(0.361)*	9.836	11.252
** Anti-parkinson drugs**	5.150	(0.266)*	0.120	(0.213)	5.030	(0.209)*	4.621	5.439
** Other anxiolytics**	3.683	(0.230)*	0.084	(0.182)	3.599	(0.183)*	3.241	3.958
** Benzodiazepines**	5.666	(1.226)*	3.804	(0.982)*	1.862	(0.955)	-0.011	3.734
** Other respiratory drugs**	0.173	(0.444)	0.038	(0.350)	0.135	(0.353)	-0.558	0.827
** General anesthetics**	0.024	(0.011)*	-0.010	(0.009)	0.034	(0.010)*	0.015	0.053
** Other hypnotics and sedatives**	0.001	(0.005)	0.009	(0.004)*	-0.008	(0.004)	-0.016	0.001
** Antimigraines**	-0.089	(0.065)	-0.014	(0.052)	-0.075	(0.051)	-0.175	0.024
** Barbiturates**	-0.183	(0.087)*	0.022	(0.069)	-0.205	(0.069)*	-0.340	-0.071
** Prokinetics**	-0.166	(0.229)	0.148	(0.181)	-0.314	(0.182)	-0.670	0.042
** Antispasmodics**	-0.834	(0.196)*	0.012	(0.155)	-0.845	(0.156)*	-1.151	-0.540
** Opioids**	-0.990	(0.303)*	0.307	(0.241)	-1.296	(0.239)*	-1.765	-0.827
** Antivertigo & antiemetics**	-2.308	(0.533)*	-0.239	(0.425)	-2.069	(0.419)*	-2.889	-1.248
** Old antihistamines**	-5.033	(0.394)*	0.247	(0.314)	-5.279	(0.310)*	-5.888	-4.673
** Central acting muscle relaxants**	-8.921	(0.594)*	1.717	(0.470)*	-10.638	(0.471)*	-11.562	-9.714

* p-value < 0.05

When the category of sedative agents was listed in order of the largest difference-in-difference value, the use of antidepressants, z-drugs, antipsychotics, antiepileptics, antiparkinsonian drugs, other anxiolytics and general anesthetics was significantly increased after index year in patients with dementia than in patients without dementia. The difference-in-difference was the highest in antidepressants (64.764 DDD/1000 patient-days) followed by Z-drugs (17.293 DDD/1000 patient-days), and antipsychotics (16.178 DDD/1000 patient-days). Also, we identified that the changes in medication use of antidepressants and antipsychotics were mainly related to agents with relatively low sedative potential, such as selective serotonin reuptake inhibitors etc. (62.467 DDD/1000 patient-days) and atypical antipsychotics (15.362 DDD/1000 patient-days). However, the use of central acting muscle relaxants, old antihistamines, antivertigo and antiemetics, opioids, antispasmodics, and barbiturates was significantly decreased after index year in patients with dementia than in patients without dementia (Table 2).

**Fig 2:**
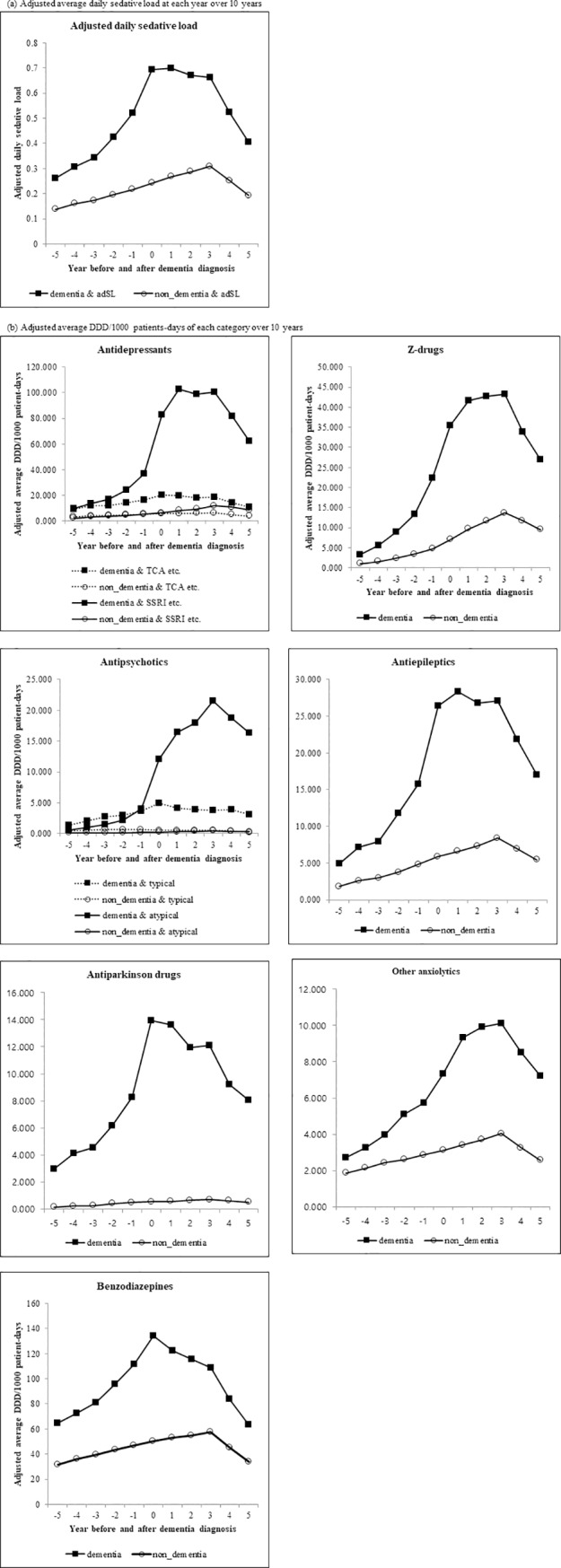
The trend of sedative load and medication use before and after diagnosis of dementia. (a) Adjusted average daily sedative load at each year over 10 years (b) Adjusted average DDD/1000 patients-days of each category over 10 years DDD; Defined daily dose, TCA; Tricyclic antidepressants, SSRI; Selective serotonin reuptake inhibitors, typical; typical antipsychotics, atypical; atypical antipsychotics.

When the adjusted adSL was evaluated by year before and after the index year, it was found to be consistently higher in patients with dementia before and after diagnosis than in those without dementia. Also, the adjusted adSL showed a gradual increase and then a tendency to decrease from 3 years after the index year in both patients with dementia and those without dementia. Among the category of sedative agents that difference-in-difference was significantly increased, this trend was similarly observed in Z-drugs, antiepileptics, antiparkinsonian drugs, and other anxiolytics. Although the difference-in-difference values of benzodiazepines was relatively low, we realized that the use of benzodiazepines before and after diagnosis was the highest in patients with dementia (64.521~111.934 DDD/1000 patient-days); it was approximately 2 times higher in patients with dementia than in patients without dementia. The trend of use of antipsychotics and antidepressants was also different with the adjusted adSL. It showed the tendency where medication use of both categories in patients with dementia steeply increased about 3~4 times at 1 year after the diagnosis, compared to the 1 year before diagnosis (Fig 2, S3 Table). The proportion of patients who were prescribed antipsychotics and antidepressants before and after diagnosis in patients with dementia was 12.9% vs. 38.7% and 38.0% vs. 55.0%, respectively (S2 Table).

## Discussion

In this study, we showed that sedative burden in patients with dementia was consistently high both before and after diagnosis, and significantly increased in patients with dementia after diagnosis compared to patients without dementia. We also showed that the trend in the use of sedative agents was different between medication categories. The use of atypical antipsychotics and antidepressants had steeply increased after dementia diagnosis. The change in benzodiazepines use was minimal, however, the proportion of its use in the overall sedative agents was greatest both before and after the diagnosis of dementia.

Benzodiazepine use was two-fold higher in patients with dementia both before and after diagnosis than in patients without dementia in the current study. Therefore, the high sedative burden in patients with dementia might be explained by the high sedative score and high use of benzodiazepine. When compared with the benzodiazepine use in overall elderly patients in Korea (66.1 DDD/1000 population/day in 2009), benzodiazepine use was found to be higher in patients with dementia, but lower in patients without dementia [25]. This difference suggests that efforts should be made to reduce the use of inappropriate benzodiazepines based on risk/benefit in patients with dementia [26]. Although we did not evaluate the relationship between benzodiazepine use and dementia onset, high use of benzodiazepines before dementia onset was in line with the result of the meta-analysis study that reported elevated odds of dementia in benzodiazepine users than in non-users (OR 1.78; 95% CI 1.33–2.38) [27]. Considering the association between higher benzodiazepine use and dementia onset in Asia in the previous study [27], further research to evaluate the relationship between sedative burden and the dementia onset in Koreans is needed.

To our knowledge, there were no studies that reported the pattern of the use of sedative agents both before and after dementia diagnosis over such a long period, so direct comparison of our results with others is difficult. The current study shows that difference-in-difference of adSL between patients with dementia and patients without dementia before and after dementia diagnosis was 0.123 unit/day. This means that the use of sedative agents with SLM score 1 after dementia onset was increased by 45 DDD/year than in patients without dementia. The tendency of an increase in sedative agent use after dementia onset was similar to that reported from a previous study that evaluated the use of anticholinergic and sedative agents each 6 months before and after dementia diagnosis [28]. The proportion of patients who were prescribed sedative agents at least once was also similar in both dementia and non-dementia cohorts, 96.1% and 96.2%, respectively. This implies that higher sedative burden in patients with dementia might be related to treatment intensity and not to patient proportion. Considering that increase in sedative burden is associated with negative clinical outcomes such as hospitalization and mortality [3, 6], more efforts may be necessary to reduce the use of sedative agents in patients with dementia.

The majority of patients with dementia used sedative agent (96.1%) in the current study, which was higher than that of previous findings (14%~85%), although direct comparison with other studies was difficult due to differences in the method of evaluating the sedative burden [12, 21, 29]. Previous studies identified medication use at specific time points using medication record or brown bag method, or carried out evaluations only when medications were used for a certain period of time. Given that we used a nationally representative claim database for evaluation of the sedative burden, the high proportion of patients who used sedative agents in our study is likely due to the inclusion of all sedative agent users even if they were given prescribed sedatives only once.

The use of antipsychotics and antidepressants steeply increased after dementia diagnosis, while sedative burden showed a steady rise from the period before dementia diagnosis. The steep elevation of antipsychotics and antidepressants may be related to the behavioral and psychological symptoms of dementia [30, 31]. In the use of antipsychotics and antidepressants, adjusted average DDD/1000 patients-days and the ratio of patients whom were prescribed medications in patients with dementia had shown similar patterns before and after diagnosis. This means that increase in the use of antipsychotics and antidepressants after dementia onset was mainly related to increase in patient proportion and not treatment intensity.

There are several limitations to consider when interpreting our results. First, the accuracy of diagnosis remains unconfirmed given that insurance claims data was used for the current study. However, we only included patients with dementia diagnosis and anti-dementia medication and excluded the patients with dementia diagnosis or anti-dementia medications during the past one year before the index date. In addition, we did not include the prescriptions of the index year for difference-in-difference estimation in order to evaluate more exactly the influence of dementia diagnosis. Second, the information of over-the-counter medications with sedative properties could not be identified in the claims data, which could lead to an underestimation of sedative burden. Third, prescription data does not reflect actual medication ingestion.

Despite those limitations, this is the first study evaluating an over 10-year use of sedative agents as medication before and after dementia diagnosis in patients compared to its use inpatients without dementia to the best of our knowledge. Also, we evaluated the sedative burden with respect to the dose and duration of sedative agents. This method could assess the sedative burden with respect to the actual pattern of medication use than the previous method that simply added the sedative score of medication ingested (or prescribed).

## Conclusion

We showed that high sedative burden in patients with dementia before and after dementia diagnosis compared to patients without dementia and sedative burden was much increased after dementia diagnosis by analyzing prescription information for a longer period.

## Supporting information

S1 Table List of sedative medications. TCA; Tricyclic antidepressants, SSRI; Selective serotonin reuptake inhibitors.
(DOCX)

S2 Table The proportion of patients who prescribed sedative agents before and after diagnosis of dementia. SSRI; Selective serotonin reuptake inhibitors.
(DOCX)

S3 Table The adjusted average DDD/1000 patient-days of sedative medication before and after diagnosis of dementia. DDD; Defined daily dose, SSRI; Selective serotonin reuptake inhibitors.
(DOCX)

## References

[1] Taipale HT, Hartikainen S, Bell JS. A comparison of four methods to quantify the cumulative effect of taking multiple drugs with sedative properties. Am J Geriatr Pharmacother. 2010;8(5):460–71. 10.1016/j.amjopharm.2010.10.004 21335299

[2] Schwertner E, Secnik J, Garcia-Ptacek S, Johansson B, Nagga K, Eriksdotter M, et al Antipsychotic Treatment Associated With Increased Mortality Risk in Patients With Dementia. A Registry-Based Observational Cohort Study. J Am Med Dir Assoc. 2019;20(3):323–9.e2. 10.1016/j.jamda.2018.12.019 30824220

[3] Kripke DF, Klauber MR, Wingard DL, Fell RL, Assmus JD, Garfinkel L. Mortality hazard associated with prescription hypnotics. Biol Psychiatry. 1998;43(9):687–93. 9583003 10.1016/s0006-3223(97)00292-8

[4] Peklar J, O'Halloran AM, Maidment ID, Henman MC, Kenny RA, Kos M. Sedative load and frailty among community-dwelling population aged >/ = 65 years. J Am Med Dir Assoc. 2015;16(4):282–9. 10.1016/j.jamda.2014.10.010 25434581

[5] Gnjidic D, Le Couteur DG, Hilmer SN, Cumming RG, Blyth FM, Naganathan V et al Sedative load and functional outcomes in community-dwelling older Australian men: the CHAMP study. Fundam Clin Pharmacol. 2014;28(1):10–9. 10.1111/j.1472-8206.2012.01063.x 22849300

[6] Yu NW, Chen PJ, Tsai HJ, Huang CW, Chiu YW, Tsay WI et al Association of benzodiazepine and Z-drug use with the risk of hospitalisation for fall-related injuries among older people: a nationwide nested case-control study in Taiwan. BMC Geriatr. 2017;17(1):140 10.1186/s12877-017-0530-4 28693443 PMC5504671

[7] Wright RM, Roumani YF, Boudreau R, Newman AB, Ruby CM, Studenski SA et al Effect of central nervous system medication use on decline in cognition in community-dwelling older adults: findings from the Health, Aging And Body Composition Study. J Am Geriatr Soc. 2009;57(2):243–50. 10.1111/j.1532-5415.2008.02127.x 19207141 PMC2744424

[8] American Geriatrics Society 2015 Updated Beers Criteria for Potentially Inappropriate Medication Use in Older Adults. J Am Geriatr Soc. 2015;63(11):2227–46. 10.1111/jgs.13702 26446832

[9] O'Mahony D, O'Sullivan D, Byrne S, O'Connor MN, Ryan C, Gallagher P. STOPP/START criteria for potentially inappropriate prescribing in older people: version 2. Age Ageing. 2015;44(2):213–8. 10.1093/ageing/afu145 25324330 PMC4339726

[10] Linjakumpu TA, Hartikainen SA, Klaukka TJ, Koponen HJ, Hakko HH, Viilo KM et al Sedative drug use in the home-dwelling elderly. Ann Pharmacother. 2004;38(12):2017–22. 10.1345/aph.1E067 15507503

[11] Linjakumpu T, Hartikainen S, Klaukka T, Koponen H, Kivela SL, Isoaho R. Psychotropics among the home-dwelling elderly—increasing trends. Int J Geriatr Psychiatry. 2002;17(9):874–83. 10.1002/gps.712 12221663

[12] Taipale HT, Bell JS, Soini H, Pitkala KH. Sedative load and mortality among residents of long-term care facilities: a prospective cohort study. Drugs Aging. 2009;26(10):871–81. 10.2165/11317080-000000000-00000 19761280

[13] Wilson NM, Hilmer SN, March LM, Cameron ID, Lord SR, Seibel MJ et al Associations between drug burden index and physical function in older people in residential aged care facilities. Age Ageing. 2010;39(4):503–7. 10.1093/ageing/afq053 20501606

[14] Parameswaran Nair N, Chalmers L, Peterson GM, Bereznicki BJ, Castelino RL, Bereznicki LR. Hospitalization in older patients due to adverse drug reactions -the need for a prediction tool. Clin Inter Aging. 2016;11:497–505. 10.2147/cia.S99097 27194906 PMC4859526

[15] Ni Chroinin D, Neto HM, Xiao D, Sandhu A, Brazel C, Farnham N et al Potentially inappropriate medications (PIMs) in older hospital in-patients: Prevalence, contribution to hospital admission and documentation of rationale for continuation. Australas J Ageing. 2016;35(4):262–5. 10.1111/ajag.12312 26970209

[16] de Mauleon A, Sourdet S, Renom-Guiteras A, Gillette-Guyonnet S, Leino-Kilpi H, Karlsson S et al Associated factors with antipsychotic use in long-term institutional care in eight European countries: Results from the RightTimePlaceCare study. J Am Med Dir Assoc. 2014;15(11):812–8. 10.1016/j.jamda.2014.06.015 25129474

[17] Wimo A, Guerchet M, Ali GC, Wu YT, Prina AM, Winblad B et al The worldwide costs of dementia 2015 and comparisons with 2010. Alzheimers Dement 2017;13(1):1–7. 10.1016/j.jalz.2016.07.150 27583652 PMC5232417

[18] Ko SJ, Jung, Y.H., Kim D.Y. The social burden and care management for people with dementia Korea Institute for Health and Social Affairs 2016;04.

[19] Zerah L, Boddaert J, Leperre-Desplanques A, Bonnet-Zamponi D, Verny M, Deligne J et al Association Between Psychotropic and Cardiovascular Iatrogenic Alerts and Risk of Hospitalizations in Elderly People Treated for Dementia: A Self-Controlled Case Series Study Based on the Matching of 2 French Health Insurance Databases. J Am Med Dir Assoc. 2017;18(6):549.e1–.e13. 10.1016/j.jamda.2017.02.001 . 28330633

[20] Linjakumpu T, Hartikainen S, Klaukka T, Koponen H, Kivela SL, Isoaho R. A model to classify the sedative load of drugs. Int J Geriatr Psychiatry. 2003;18(6):542–4. 10.1002/gps.846 12789678

[21] Parsons C, Haydock J, Mathie E, Baron N, Machen I, Stevenson E et al Sedative load of medications prescribed for older people with dementia in care homes. BMC Geriatr. 2011;11:56 10.1186/1471-2318-11-56 21958366 PMC3197480

[22] Jean-Bart E, Moutet C, Dauphinot V, Krolak-Salmon P, Mouchoux C. Exposure to anticholinergic and sedative medicines as indicators of high-risk prescriptions in the elderly. Int J Clin Pharm. 2017;39(6):1237–47. 10.1007/s11096-017-0533-4 29086145

[23] Austin PC. Optimal caliper widths for propensity-score matching when estimating differences in means and differences in proportions in observational studies. Pharm Stat. 2011;10(2):150–61. 10.1002/pst.433 20925139 PMC3120982

[24] Norgaard A, Jensen-Dahm C, Gasse C, Hansen ES, Waldemar G. Psychotropic Polypharmacy in Patients with Dementia: Prevalence and Predictors. J Alzheimer Dis. 2017;56(2):707–16. 10.3233/JAD-160828 28035931

[25] Hwang SH, Han S, Choi H, Park C, Kim SM, Kim TH. Trends in the prescription of benzodiazepines for the elderly in Korea. BMC Psychiatry. 2017;17(1):303 10.1186/s12888-017-1467-z 28830488 PMC5567896

[26] Lapeyre-Mestre M. A Review of Adverse Outcomes Associated with Psychoactive Drug Use in Nursing Home Residents with Dementia. Drugs Aging. 2016;33(12):865–88. 10.1007/s40266-016-0414-x 27812994

[27] Islam MM, Iqbal U, Walther B, Atique S, Dubey NK, Nguyen PA et al Benzodiazepine Use and Risk of Dementia in the Elderly Population: A Systematic Review and Meta-Analysis. Neuroepidemiology. 2016;47(3–4):181–91. 10.1159/000454881 28013304

[28] Gadzhanova S, Roughead E, Robinson M. Use of Medicines with Anticholinergic and Sedative Effect Before and After Initiation of Anti-Dementia Medications. Drugs Real World Outcomes. 2015;2(1):53–60. 10.1007/s40801-015-0012-y 27747617 PMC4883199

[29] Hilmer SN, Mager DE, Simonsick EM, Cao Y, Ling SM, Windham BG et al A drug burden index to define the functional burden of medications in older people. Arch Intern Med. 2007;167(8):781–7. 10.1001/archinte.167.8.781 17452540

[30] Reus VI, Fochtmann LJ, Eyler AE, Hilty DM, Horvitz-Lennon M, Jibson MD et al The American Psychiatric Association Practice Guideline on the Use of Antipsychotics to Treat Agitation or Psychosis in Patients With Dementia. Am J Psychiatry. 2016;173(5):543–6. 10.1176/appi.ajp.2015.173501 27133416

[31] Herrmann N, Gauthier S, Lysy PG. Clinical practice guidelines for severe Alzheimer's disease. Alzheimers Dement. 2007;3(4):385–97. 10.1016/j.jalz.2007.07.007 19595959

