# Supplementary material for: Change in sedative burden after dementia onset using difference-in-difference estimations
Source: PLoS One. 2019 Aug 2;14(8):e0220582. doi: 10.1371/journal.pone.0220582 (PMC6677315; doi:10.1371/journal.pone.0220582)
Supplement: S1 Table — TCA; Tricyclic antidepressants, SSRI; Selective serotonin reuptake inhibitors. (DOCX) [file pone.0220582.s001.docx]

**S1 Table. List of sedative medications**

| **Category** | **Examples of medicines** |
| --- | --- |
| *Group 1 (Primary sedatives, Score 2)* | |
| Antidepressants ; TCA etc. | Amitriptyline, amoxapine, clomipramine, dothiepin, doxepin, imipramine, mianserine, moclobemide, nortriptyline, quinupramine, trazodone |
| Barbiturates | Pentobarbital, phenobarbital, thiamylal, thiopental |
| Benzodiazepines | Alprazolam, bromazepam, brotizolam, chlordiazepoxide, clobazam, clonazepam, clorazepate, clotiazepam, diazepam, estazolam, ethyl loflazepate, etizolam, flunitrazepam, flurazepam lorazepam, mexazolam, midazolam, pinazepam, temazepam, tofisopam, triazolam |
| General anesthetics | Etomidate, ketamine, propofol |
| Other anxiolytics | Buspirone, hydroxyzine, tandospirone |
| Other hypnotic and sedatives | Chloral hydrate, dichloraphenazone |
| Traditional antipsychotics | Bromperidol, chlorpromazine, chlorprothixene, droperidol, haloperidol, levomepromazine, lithium, mesoridazine, molindone, nemonapride, perphenazine, pimozide, sulpiride, thioridazine, thiothixene, tiapride, trifluoperazine, zotepine, zuclopenthixol |
| Z-drugs | Zolpidem, zopiclone |
|  |  |
| *Group 2 (Drugs with sedation as a prominent side effect or preparations with a sedating component, Score 1)* | |
| Antidepressants; SSRI etc. | Bupropion, citalopram, duloxetine, escitalopram, fluoxetine, fluvoxamine, hyperici herba, medifoxamine fumarate, milnacipran, mirtazapine, nefazodone, paroxetine, sertraline, tianeptine, toloxatone, venlafaxine |
| Antiepileptics | Carbamazepine, fosphenytoin, gabapentin, lamotrigine, levetiracetam, oxcarbazepine, phenytoin, pregabalin, primidone, topiramate, valproic acid, vigabatrin, zonisamide |
| Antimigraine | Almotriptan, ergotamine, frovatriptan, naratriptan, sumatriptan, zolmitriptan |
| Antiparkinson drugs | Benzatropine, biperiden, procyclidine, trihexyphenidyl |
| Antivertigo & antiemetics | Betahistine, cinnarizine, difenidol, dimenhydrinate |
| Atypical antipsychotics | Amisulpride, aripiprazole, blonanserin, clozapine, olanzapine, paliperidone, quetiapine, risperidone, ziprasidone |
| Central muscle relaxants | Afloqualone, baclofen, carisoprodol, chlormezanone, chlorphenesin, chlorzoxazone, cyclobenzaprine, eperisone, methocarbamol, orphenadrine, pridinol, thiocolchicoside, tizanidine, tolperisone |
| Opioids | Alfentanil, buprenorphine, butorphanol, codeine, dihydrocodeine, fentanyl, hydrocodone, hydromorphone, morphine, nalbuphine, oxycodone, oxycodone and naloxone, pentazocine, pethidine, remifentanil, sufentanil, tramadol |
| Old antihistamines | Alimemazine, brompheniramine, buclizine, carbinoxamine, chlorcinnazine, chlorpheniramine, clemastine, cyproheptadine, dexbrompheniramine, diphenhydramine, diphenylpyraline, doxylamine, homochlorcyclizine, mequitazine, oxatomide, oxomemazine, pheniramine, piprinhydrinate, triprolidine, promethazine |
| Prokinetics | Metoclopramide |
| Respiratory | Benproperine, benzonatate, bromhexine, dextromethorphan, levodropropizine |
