# Supplementary material for: Change in sedative burden after dementia onset using difference-in-difference estimations
Source: PLoS One. 2019 Aug 2;14(8):e0220582. doi: 10.1371/journal.pone.0220582 (PMC6677315; doi:10.1371/journal.pone.0220582)
Supplement: S2 Table — SSRI; Selective serotonin reuptake inhibitors. (DOCX) [file pone.0220582.s002.docx]

**S2 Table. The proportion of patients who was prescribed sedative agents before and after diagnosis of dementia**

|  |  | **Before**  **dementia diagnosis**  **(N=10,833)** | | **After**  **dementia diagnosis**  **(N=43,332)** | |
| --- | --- | --- | --- | --- | --- |
|  |  | **N** | **(%)** | **N** | **(%)** |
| **Overall sedatives** | dementia | 10231 | (94.4) | 10418 | (96.2) |
|  | non-dementia | 40377 | (93.2) | 41638 | (96.1) |
| **Antidepressants** | dementia | 4116 | (38.0) | 5955 | (55.0) |
|  | non-dementia | 9529 | (22.0) | 11822 | (27.3) |
| SSRI etc. | dementia | 1718 | (15.9) | 3866 | (35.7) |
|  | non-dementia | 2149 | (5.0) | 5036 | (11.6) |
| Tricyclic agents etc. | dementia | 3469 | (32.0) | 4160 | (38.4) |
|  | non-dementia | 8583 | (19.8) | 9217 | (21.3) |
| **Z-drug** | dementia | 1689 | (15.6) | 3559 | (32.9) |
|  | non-dementia | 3063 | (7.1) | 7678 | (17.7) |
| **Antipsychotics** | dementia | 1395 | (12.9) | 4193 | (38.7) |
|  | non-dementia | 1817 | (4.2) | 1704 | (3.9) |
| Atypical antipsychotics | dementia | 519 | (4.8) | 3578 | (33.0) |
|  | non-dementia | 137 | (0.3) | 598 | (1.4) |
| Traditional antipsychotics | dementia | 1133 | (10.5) | 1742 | (16.1) |
|  | non-dementia | 1730 | (4.0) | 1283 | (3.0) |
| **Antiepileptics** | dementia | 2295 | (21.2) | 3078 | (28.4) |
|  | non-dementia | 5824 | (13.4) | 8049 | (18.6) |
| **Anti-parkinson drugs** | dementia | 436 | (4.0) | 926 | (8.5) |
|  | non-dementia | 217 | (0.5) | 307 | (0.7) |
| **Other anxiolytics** | dementia | 3349 | (30.9) | 3360 | (31.0) |
|  | non-dementia | 12406 | (28.6) | 13753 | (31.7) |
| **Benzodiazepines** | dementia | 8392 | (77.5) | 8217 | (75.9) |
|  | non-dementia | 30428 | (70.2) | 31197 | (72.0) |
| **Other respiratory drugs** | dementia | 5682 | (52.5) | 5336 | (49.3) |
|  | non-dementia | 22998 | (53.1) | 25280 | (58.3) |
| **General anesthetics** | dementia | 327 | (3.0) | 621 | (5.7) |
|  | non-dementia | 1051 | (2.4) | 2370 | (5.5) |
| **Other hypnotics and sedatives** | dementia | 74 | (0.7) | 11 | (0.1) |
|  | non-dementia | 236 | (0.5) | 31 | (0.1) |
| **Antimigraines** | dementia | 333 | (3.1) | 235 | (2.2) |
|  | non-dementia | 908 | (2.1) | 937 | (2.2) |
| **Barbiturates** | dementia | 687 | (6.3) | 523 | (4.8) |
|  | non-dementia | 2339 | (5.4) | 1851 | (4.3) |
| **Prokinetics** | dementia | 4329 | (40.0) | 4950 | (45.7) |
|  | non-dementia | 14427 | (33.3) | 19695 | (45.5) |
| **Antispasmodics** | dementia | 3649 | (33.7) | 2725 | (25.2) |
|  | non-dementia | 13894 | (32.1) | 13244 | (30.6) |
| **Opioids** | dementia | 7636 | (70.5) | 8364 | (77.2) |
|  | non-dementia | 29950 | (69.1) | 36909 | (85.2) |
| **Antivertigo & antiemetics** | dementia | 3390 | (31.3) | 2911 | (26.9) |
|  | non-dementia | 10045 | (23.2) | 13021 | (30.0) |
| **Old antihistamines** | dementia | 8920 | (82.3) | 8456 | (78.1) |
|  | non-dementia | 36420 | (84.0) | 37431 | (86.4) |
| **Central acting muscle relaxants** | dementia | 7612 | (70.3) | 6525 | (60.2) |
|  | non-dementia | 29695 | (68.5) | 31377 | (72.4) |
