# Supplementary material for: Change in sedative burden after dementia onset using difference-in-difference estimations
Source: PLoS One. 2019 Aug 2;14(8):e0220582. doi: 10.1371/journal.pone.0220582 (PMC6677315; doi:10.1371/journal.pone.0220582)
Supplement: S3 Table — DDD; Defined daily dose, SSRI; Selective serotonin reuptake inhibitors. (DOCX) [file pone.0220582.s003.docx]

**S3 Table. The adjusted average DDD/1000 patient-days of sedative medication before and after diagnosis of dementia**

| **Year of before and after dementia diagnosis** |  | **-5** | **-4** | **-3** | **-2** | **-1** | **0** | **1** | **2** | **3** | **4** | **5** |
| --- | --- | --- | --- | --- | --- | --- | --- | --- | --- | --- | --- | --- |
| **N** | dementia | 5,386 | 6,971 | 8,302 | 9,295 | 10,096 | 10,833 | 10,620 | 10,407 | 9,846 | 8,916 | 7,250 |
|  | non-dementia | 22,287 | 29,092 | 34,089 | 37,396 | 39,980 | 40,601 | 40,838 | 40,902 | 40,136 | 38,494 | 32,231 |
| **Antidepressants** |  |  |  |  |  |  |  |  |  |  |  |  |
| SSRI etc. | dementia | 9.728 | 13.567 | 17.315 | 24.266 | 36.709 | 82.924 | 102.806 | 99.103 | 100.52 | 81.961 | 62.365 |
|  | non-dementia | 2.377 | 3.237 | 3.823 | 4.485 | 5.502 | 6.375 | 8.334 | 9.542 | 11.88 | 10.815 | 8.484 |
| Tricyclic agents etc. | dementia | 9.213 | 11.796 | 12.014 | 14.01 | 16.323 | 20.357 | 19.565 | 18.356 | 18.578 | 14.318 | 10.76 |
|  | non-dementia | 3.232 | 4.103 | 4.6 | 5.062 | 5.39 | 5.795 | 5.93 | 6.185 | 6.5 | 5.045 | 3.82 |
| **Z-drug** | dementia | 3.381 | 5.644 | 8.904 | 13.375 | 22.422 | 35.488 | 41.688 | 42.761 | 43.261 | 33.871 | 26.911 |
|  | non-dementia | 1.018 | 1.552 | 2.332 | 3.474 | 4.745 | 7.022 | 9.724 | 11.638 | 13.666 | 11.774 | 9.55 |
| **Antipsychotics** |  |  |  |  |  |  |  |  |  |  |  |  |
| Atypical antipsychotics | dementia | 0.516 | 1.057 | 1.51 | 2.231 | 4.07 | 12.09 | 16.461 | 17.917 | 21.545 | 18.8 | 16.319 |
|  | non-dementia | 0.12 | 0.104 | 0.136 | 0.155 | 0.16 | 0.22 | 0.296 | 0.374 | 0.501 | 0.383 | 0.307 |
| Traditional antipsychotics | dementia | 1.391 | 2.053 | 2.734 | 2.96 | 3.647 | 4.929 | 4.139 | 3.906 | 3.81 | 3.954 | 3.116 |
|  | non-dementia | 0.408 | 0.569 | 0.72 | 0.665 | 0.635 | 0.516 | 0.534 | 0.523 | 0.507 | 0.395 | 0.212 |
| **Antiepileptics** | dementia | 4.978 | 7.146 | 7.976 | 11.814 | 15.782 | 26.374 | 28.356 | 26.821 | 27.07 | 21.829 | 16.983 |
|  | non-dementia | 1.803 | 2.574 | 2.977 | 3.747 | 4.825 | 5.866 | 6.622 | 7.326 | 8.38 | 6.946 | 5.449 |
| **Anti-parkinson drugs** | dementia | 2.941 | 4.107 | 4.533 | 6.172 | 8.263 | 13.94 | 13.623 | 11.923 | 12.116 | 9.23 | 8.05 |
|  | non-dementia | 0.152 | 0.209 | 0.259 | 0.398 | 0.477 | 0.527 | 0.562 | 0.647 | 0.7 | 0.624 | 0.504 |
| **Other anxiolytics** | dementia | 2.728 | 3.259 | 3.965 | 5.11 | 5.735 | 7.327 | 9.339 | 9.935 | 10.116 | 8.511 | 7.207 |
|  | non-dementia | 1.864 | 2.138 | 2.416 | 2.6 | 2.852 | 3.101 | 3.413 | 3.689 | 4.04 | 3.244 | 2.554 |
| **Benzodiazepines** | dementia | 64.521 | 72.625 | 80.868 | 95.95 | 111.934 | 134.229 | 122.526 | 115.623 | 108.84 | 83.761 | 63.721 |
|  | non-dementia | 31.825 | 36.353 | 39.271 | 43.288 | 46.99 | 50.269 | 53.158 | 55.1 | 57.419 | 45.405 | 34.165 |
| **Other respiratory drugs** | dementia | 6.945 | 7.329 | 7.846 | 8.676 | 11.865 | 14.398 | 15.082 | 15.463 | 17.238 | 14.379 | 10.519 |
|  | non-dementia | 5.585 | 6.410 | 6.366 | 7.246 | 8.066 | 10.129 | 12.213 | 13.504 | 14.923 | 12.744 | 9.757 |
| **General anesthetics** | dementia | 0.002 | 0.008 | 0.007 | 0.016 | 0.029 | 0.080 | 0.071 | 0.054 | 0.045 | 0.036 | 0.090 |
|  | non-dementia | 0.002 | 0.006 | 0.006 | 0.015 | 0.011 | 0.026 | 0.017 | 0.022 | 0.020 | 0.016 | 0.024 |
| **Other hypnotics and sedatives** | dementia | 0.044 | 0.010 | 0.012 | 0.018 | 0.005 | 0.009 | 0.006 | 0.007 | 0.001 | 0.000 | 0.000 |
|  | non-dementia | 0.019 | 0.015 | 0.012 | 0.008 | 0.006 | 0.004 | 0.007 | 0.007 | 0.006 | 0.000 | 0.001 |
| **Antimigraines** | dementia | 0.254 | 0.443 | 0.600 | 0.603 | 0.534 | 0.571 | 0.528 | 0.414 | 0.517 | 0.590 | 0.465 |
|  | non-dementia | 0.149 | 0.201 | 0.182 | 0.197 | 0.230 | 0.222 | 0.237 | 0.214 | 0.224 | 0.179 | 0.141 |
| **Barbiturates** | dementia | 0.548 | 0.807 | 0.546 | 0.789 | 0.885 | 1.230 | 0.625 | 0.498 | 0.338 | 0.373 | 0.366 |
|  | non-dementia | 0.391 | 0.451 | 0.424 | 0.446 | 0.369 | 0.450 | 0.359 | 0.350 | 0.274 | 0.209 | 0.138 |
| **Prokinetics** | dementia | 4.079 | 4.572 | 4.321 | 4.906 | 6.080 | 7.858 | 6.727 | 5.902 | 5.109 | 4.135 | 3.209 |
|  | non-dementia | 2.525 | 2.766 | 3.195 | 3.505 | 3.747 | 3.887 | 4.178 | 4.183 | 4.049 | 3.404 | 2.643 |
| **Antispasmodics** | dementia | 4.003 | 3.490 | 3.591 | 3.785 | 3.409 | 3.111 | 2.448 | 1.857 | 1.940 | 1.304 | 0.585 |
|  | non-dementia | 2.999 | 3.282 | 3.063 | 3.147 | 3.191 | 2.813 | 2.608 | 2.430 | 2.235 | 1.580 | 1.108 |
| **Opioids** | dementia | 3.570 | 4.282 | 5.069 | 7.083 | 10.473 | 13.475 | 14.056 | 15.071 | 16.039 | 12.784 | 9.955 |
|  | non-dementia | 2.181 | 2.920 | 3.453 | 4.568 | 5.844 | 7.961 | 10.499 | 13.042 | 15.366 | 13.035 | 10.274 |
| **Antivertigo & antiemetics** | dementia | 11.172 | 12.708 | 13.863 | 19.528 | 23.304 | 27.051 | 24.639 | 22.968 | 21.888 | 17.196 | 12.683 |
|  | non-dementia | 5.429 | 6.355 | 7.085 | 8.151 | 9.114 | 10.774 | 11.922 | 13.529 | 14.982 | 12.593 | 9.812 |
| **Old antihistamines** | dementia | 19.367 | 21.223 | 21.746 | 22.894 | 23.345 | 22.263 | 20.228 | 19.417 | 18.843 | 15.221 | 11.461 |
|  | non-dementia | 17.459 | 18.787 | 19.139 | 20.875 | 20.794 | 21.257 | 21.752 | 22.406 | 22.834 | 18.494 | 14.031 |
| **Central acting muscle relaxants** | dementia | 19.141 | 22.704 | 23.724 | 29.037 | 34.014 | 36.109 | 33.249 | 29.771 | 27.708 | 21.074 | 14.808 |
|  | non-dementia | 14.524 | 16.019 | 17.342 | 20.167 | 23.730 | 27.567 | 30.083 | 32.162 | 33.411 | 26.653 | 20.104 |
